# Development of Rapid Immunochromatographic Test for Hemagglutinin Antigen of H7 Subtype in Patients Infected with Novel Avian Influenza A (H7N9) Virus

**DOI:** 10.1371/journal.pone.0092306

**Published:** 2014-03-19

**Authors:** Keren Kang, Li Chen, Xiang Zhao, Chengfeng Qin, Zanwu Zhan, Jihua Wang, Wenmei Li, Emmanuel E. Dzakah, Weijuang Huang, Yuelong Shu, Tao Jiang, Wuchun Cao, Mingquan Xie, Xiaochun Luo, Shixing Tang

**Affiliations:** 1 School of Bioscience and Bioengineering, South China University of Technology, Guangzhou, China; 2 National Engineering Laboratory of Point-of-Care Tests, Guangzhou Wondfo Biotech Co. Ltd, Guangzhou, China; 3 Chinese Center for Disease Control and Prevention, Beijing, China; 4 Beijing Institute of Microbiology and Epidemiology, Beijing, China; Centers for Disease Control and Prevention, United States of America

## Abstract

**Background:**

Since human infection with the novel H7N9 avian influenza virus was identified in China in March 2013, the relatively high mortality rate and possibility of human-to-human transmission have highlighted the urgent need for sensitive and specific assays for diagnosis of H7N9 infection.

**Methodology/Principal Findings:**

We developed a rapid diagnostic test for the novel avian influenza A (H7N9) virus using anti-hemagglutinin (HA) monoclonal antibodies specifically targeting H7 in an immunochromatographic assay system. The assay limit of detection was 10^3.5^ pfu/ml or 10^3^TCID_50_ of H7N9 virus. The assay specifically detected H7N9 viral isolates and recombinant HA proteins of H7 subtypes including H7N7 and H7N9, but did not react with non-H7 subtypes including H1N1, H3N2, H5N1, H5N9, and H9N2. The detection sensitivity was 59.4% (19/32) for H7N9 patients confirmed by RT-PCR. Moreover, the highest sensitivity of 61.5% (16/26) was obtained when testing H7N9 positive sputum samples while 35.7% (5/14) of nasopharyngeal swabs and 20% (2/10) of fecal samples tested positive. No false positive detection was found when testing 180 H7N9 negative samples.

**Conclusions/Significance:**

Our novel rapid assay can specifically detect H7 HA antigen, facilitating rapid diagnosis for prevention and control of the on-going H7N9 epidemic.

## Introduction

The novel H7N9 avian influenza virus was first isolated from three patients with severe lower respiratory tract disease of unknown cause in China in March 2013 [1]. As of November 6^th^, 2013, a total of 139 cases of H7N9 infection had been laboratory-confirmed, causing 45 deaths in mainland China and 1 in Taiwan [2], [3]. As of January 28 of 2014, more than 100 new H7N9 cases have been reported. H7N9 was initially detected in the Yangtze River Delta, and area encompassing Shanghai, Zhejiang, Anhui and Jiangsu provinces, but has since spread to more than 12 provinces and cities including Beijing, Hebei, Shandong, Henan, Jiangxi, Hunan, Fujian, Guangdong and Taiwan, indicating a continuing epidemic of H7N9 infection in humans. Severe lower respiratory tract infection was predominant in human H7N9 infection, and a relatively high mortality of about 32.4% (45/139) was observed as of November6^th^, 2013 [1], [2], [4], [5].

Human infection with subtype H7 avian influenza A viruses had rarely been described and never reported in China before 2013. These infections may have been overlooked due to mild symptoms or asymptomatic infection in humans and low pathogenicity in poultry [6]–[9]. Before the 2013 outbreak of H7N9, a highly pathogenic avian influenza A(H7N7) infection was reported to result in one fatality in the Netherlands in 2003 [10], [11].

The novel H7N9 virus is a reassortant of triple avian influenza viruses and has genetic markers that can bind strongly to human-like receptors, and are known to be associated with improved replication of avian influenza viruses in mammals [1], [4], [6]. Therefore, the possibility of human-to-human transmission cannot be ruled out [12]. Although no sustained human-to-human transmission of H7N9 virus has been confirmed [1]–[4], one case of transmission between a father and daughter has been identified in Eastern China by genetic comparison of viral isolates [12].

The hemagglutinin gene sequence suggests that the novel H7N9 virus may be a low pathogenic avian influenza virus, and has been found in poultry including pigeons and chickens [4]. However, the extent of distribution of asymptomatic infection of H7N9 virus in human and domestic poultry is unclear partly due to the lack of well-evaluated testing kits.

Diagnosis of H7N9 infection relies on isolation and culture of H7N9 virus from patient samples, requiring a biosafety level 3 laboratory equipped with real-time reverse-transcription PCR analysis (RT-PCR) capabilities, and well trained technical personnel. Real-time RT-PCR assays for specific detection of H7N9 virus were established shortly after the initial outbreak [13], and in May and July, 2013 the Chinese Food and Drug Administration (CFDA) approved three PCR kits for specific detection of H7N9 virus [14]. However, so far no well-evaluated rapid immunoassays have been approved. Considering the high mortality rate and the possibility of human-to-human transmission of the novel H7N9 virus, a simple rapid diagnostic screening assay is critical for monitoring this epidemic in both humans and animals. Such a test is urgently required for rapid diagnosis and early antiviral treatment of patients infected with H7N9, and may facilitate detection of asymptomatic infections. Here we report the development and evaluation of a simple lateral flow assay for rapid detection of hemagglutinin (HA) H7 subtype antigen.

## Materials and Methods

### Ethics Statement

The study protocol and informed consent documents were reviewed and approved by the Ethics Committee of the First Affiliated Hospital, College of Medicine, Zhejiang University. All participants provided written informed consent.

### Monoclonal Antibodies and Influenza Virus Proteins

The H7-directed monoclonal antibodies C1 (clone 1175-18C4) and C2 (clone 2113-3B7), and recombinant HA proteins of influenza A viruses H1N1, H3N2, H5N1, H7N7 and H7N9 (A/Shanghai/2/2013) were purchased from Immune Technology Corp. (NY, USA). Monoclonal anti-H7 antibodies were obtained by DNA immunization of animals with plasmid DNA containing the full length of HA gene of H7N7 virus (H7N7/England/268/96) (Genebank No. AF028020). In addition, recombinant HA proteins of H7N9 (A/Anhui/1/2013) and H7N7 were kindly provided by Dr. Ling Chen of the State Key Laboratory of Respiratory Diseases (Guangzhou, China) while inactivated H1N1, H5N9, and H9N2 virus lysates were obtained from South China University of Technology (Guangzhou, China).

### Influenza Viruses

Influenza A (H7N9) (A/Anhui/1/2013) was kindly provided by the Institute of Microbiology and Epidemiology, Academy of Military Medical Sciences (Beijing, China). Another 4 H7N9 virus isolates were provided by the State Key Laboratory for Diagnosis and Treatment of Infectious Diseases, First Affiliated Hospital, College of Medicine, Zhejiang University (Hangzhou, China). All H7N9 viruses were obtained by propagating clinical specimens in the allantoic sac and amniotic cavity of 9-to-11-day-old specific pathogen-free embryonated chicken eggs for 48 to 72 h at 35°C. The virus titers (TCID_50_ or pfu/ml) of H7N9 samples were determined as described earlier [15].

### Quality Control Panel

The quality control panel for the H7N9 immunoassay was created by the National Institute for Viral Disease Control and Prevention, Chinese Center for Disease Control and Prevention, Beijing, China and consisted of 7 dilution samples from one H7N9 positive sample and 20 H7N9 negative samples.

### Clinical Samples

A total of 99 samples including throat swabs, sputum and feces were collected from 32 patients with laboratory-confirmed H7N9 infections. The average age of these patients was 62±16 years (ranging from 36 to 86 years), and 62.5% (20/32) were male. In addition, throat swab and sputum samples were also collected from 180 patients with fever or influenza-like symptoms that were not infected with H7N9. The average age of these patients was 43±17 years (ranging from 21 to 81 years), and 48.9% were male. Of the H7N9 negative patients, twelve suffered from influenza, including 7 patients with confirmed H1N1 infection. Six H7N9 negative patients tested positive for measles, 10 patients for TB, and 15 patients for mycoplasma.

### Lateral Flow Immunoassay

H7 directed C1and C2 monoclonal antibodies were used as capture and detector antibodies respectively in the lateral flow immunoassay. C2 (20 ug/ml) was conjugated with 0.01% colloidal gold and sprayed on glass fiber. C1 (0.8 mg/ml) and goat-anti-mouse IgG (1.0 mg/ml, purchased from Abcam, Cambridge, MA, USA) were sprayed on a nitrocellulose membrane to form the test and control lines, respectively. There was no cross-reactivity between the anti-mouse IgG and human IgG. The glass fiber pad and membrane were left to dry at 30°C overnight. The nitrocellulose membrane and glass fiber membrane were assembled into a lateral flow immunoassay strip. The assembly was comprised of 4.0 mm wide strips inserted into a cassette. The purified proteins or clinical samples were first diluted in phosphate-buffered saline (PBS) with 0.025% Tween 20 and 0.1% BSA (PBS-T), and then applied to the sample hole of the test cassettes. Throat swab samples were immersed in 400 μl PBS-T and 60 μl of the solution was applied to the sample hole on the test cassette. For sputum samples, 20 μl samples were dissolved in 60 μl of dilution buffer and 60 μl of the resultant solution was applied to the sample hole of the test cassette. Fecal samples (about 0.2 g) were also dissolved in 400 μl dilution buffer, centrifuged at 17000 g for 2 min and 60 μl of the resultant supernatant was applied to the test cassette. The result of the lateral flow assay was obtained within 15 min. Double red lines in the reading windows indicated a positive result. A red control line alone indicated a negative result. If control line did not appear within 15 min, the test was considered invalid. The signal intensity of the test lines was compared with a standard color chat consisting of 9 grades from C1 to C9. Weak (+), moderate (++) and strong (+++) positivity represent signal intensity of C7 to C9, C4 to C6 and C1 to C3, respectively.

### Real-time RT-PCR

Real-time RT-PCR was carried out using primers and protocols previously described [13]. Briefly, a 25 μl reaction was set up containing 5 μl of template RNA, 12.5 μl of 2 X RT-PCR master mix, 1 μl of 25 X RT-PCR enzyme mix, 0.5 μl of Probe (20 μM), 0.5 μl of each of the primers (40 μM) and 5 μl of RNase-free water. Thermal cycling conditions consisted of 45°C for 10 min, followed by 95°C for 10 min and then 40 cycles of 95°C for 15 s, 60°C for 45 s. H7N9 viral RNA and non-H7N9 viral RNA were used as positive and negative controls, respectively while RNase free water was used as blank control.

## Results

### Analytic Sensitivity and Specificity

We created a panel of recombinant HA proteins with which to determine the specificity of our novel lateral flow immunoassay rapid diagnostic test (RDT). The panel included recombinant HA derived from two N7N9 viruses, A/Shanghai/2/2013 and A/Anhui/1/2013; one H7N7 virus A/Netherlands/219/03; one H5N1 virus, A/Hubei/1/2010; one H3N2 virus, A/Victoria/361/2011; and one H1N1 virus, A/California/06/2009. The limit of detection (LOD) for H7N9 virus HA was 1 ng/ml, and for the H7N7 virus was 10 ng/ml (Table 1). No cross-reactions were observed when testing 1000 ng/ml recombinant HA proteins of H1N1, H3N2, H5N1 (Table 1), or 1000 ng/ml of viral lysates of H1N1, H5N9 and H9N2 viruses (data not shown).

**Table 1:** Analytic sensitivity and specificity of rapid diagnostic test for recombinant hemagglutinin derived from influenza viruses.

Proteins tested	Virus strains	Concentration(ng/ml)	RDT results*
Recombinant HA	A/Shanghai/2/2013(H7N9)	1	+
		10	++
		100	+++
		1000	+++
Recombinant HA	A/Anhui/1/2013(H7N9)	1	+
		10	++
		100	+++
		1000	+++
Recombinant HA	A/Netherlands/2/19/03	1	−
		10	+
		100	++
		1000	+++
Recombinant HA	A/Hubei/1/2010(H5N1)	1000	−
Recombinant HA	A/Victoria/361/2011(H3N2)	1000	−
Recombinant HA	A/California/06/2009(H1N1)	1000	−

*Signal intensity was determined by comparing with a standard color chart and categorized as weak (+, C7–9), moderate (++, C4–6) or strong (+++, C1–3) positivity, respectively. “−” indicates a negative test result.

The analytic sensitivity and specificity of the novel RDT was further probed using a quality control panel including a series of dilution of one H7N9 positive sample and 20 H7N9 negative clinical samples (Table 2). All 7 dilutions of H7N9 sample tested positive by our RDT, and no false positive results were observed for the 20 H7N9 negative samples including inactive H1N1, H3N2 and influenza B viruses (Table 2).

**Table 2:** Rapid Diagnostic Test Quality Control Panel.

Code	Virus strains	Subtypes	Virus titer (TCID_50_)	HA titer	RDT results*
P1	A/Anhui/1/2013	H7N9	10^7^		+++
P2	A/Anhui/1/2013	H7N9	10^6^		+++
P3	A/Anhui/1/2013	H7N9	10^5^		++
P4	A/Anhui/1/2013	H7N9	10^4^		+
P5	A/Anhui/1/2013	H7N9		128	+++
P6	A/Anhui/1/2013	H7N9		12.8	++
P7	A/Anhui/1/2013	H7N9		1.28	+
N1	A/California/7/2009	H1N1Pdm		32	−
N2	A/Perth/16/2009	H3N2		128	−
N3	B/Chongqing Yuzhong/1384/2010	BV		128	−
N4	B/Guangdong Luohu/1512/2010	BY		128	−
N5	A/Chongqing Yuzhong/1_50_/2007	H1N1		32	−
N6	B/Heilongjiang Hulan/116/2010	BV		16	−
N7	B/Shanghai Luwan/173/2011	BY		32	−
N8	B/Guangdong Luohu/1512/2010	BY		128	−
N9	B/Shanxi Beiling/127/2008	BY		16	−
N10	B/Gansu Chengguan/1118/2008	BY		64	−
N11	B/Hubei Wujiagang/158/2009	BV		32	−
N12	A/Fujian Tongan/196/2009	H3N2		128	−
N13	A/Brisbane/10/2007	H3N2		128	−
N14	A/Hubei Jiangan/1139/2009	H3N2		32	−
N15	A/Yunnan/1145/2005	H3N2		128	−
N16	A/Hubei Beihu/1143/2011	H3N2		64	−
N17	A/Liaoning Huanggu/1183/2007	H1N1		256	−
N18	A/Jiangxi Donghu/312/2006	H3N2		128	−
N19	A/Anhui Baohe/137/2008	H3N2		64	−
N20	A/Guangdong Nongan/SWL112/2010	H1N1Pdm		128	−

* Signal intensity was determined by comparing with a standard color chart and categorized as weak (+, C7–9), moderate (++, C4–6) or strong (+++, C1–3) positivity, respectively. “−” indicates a negative test result.

The LOD of our RDT was further characterized by testing a series of diluted H7N9 virus culture supernatants with known TCID_50_ or pfu/ml (Table 3). Samples with low levels of H7N9 virus (10^3^TCID_50_ or 10^3.5^pfu/ml) tested positive by our assay (Table 3).

**Table 3:** Detection Limit of Rapid Diagnostic Test and Real Time RT-PCR for H7N9 isolates.

H7N9 virus isolate	Source	Dilution titers	Labs tested	LOD of RDT	Ct of RT-PCR
Isolate 1	Zhejiang	10^−1^∼10^−5^	Zhejiang^a^	10^−5^	31.36
Isolate 2	Zhejiang	10^−1^∼10^−5^	Zhejiang^a^	10^−4^	27.21
Isolate 3	Zhejiang	10^−1^∼10^−5^	Zhejiang^a^	10^−5^	29.67
Isolate 4	Zhejiang	10^−1^∼10^−5^	Zhejiang^a^	10^−4^	26.52
A/Anhui/1/2013	Anhui	10^−1^∼10^−7^TCID_50_	Beijing #1^b^	10^3^TCID_50_	30.00
A/Anhui1/1/2013	Anhui	10^0.5^∼10^5.5^ pfu/ml	Beijing #2^c^	10^3.5^ pfu/ml	29.00
A/Shanghai1/1/2013	Shanghai	10^0.5^∼10^5.5^ pfu/ml	Beijing #2^c^	10^3.5^ pfu/ml	29.00

a State Key Laboratory for Diagnosis and Treatment of Infection Diseases, First Affiliated Hospital, College of Medicine, Zhejiang University.

b Beijing #1: Influenza Branch of the Center for Disease Prevention and Control of China.

c Beijing #2: Institute of Microbiology and Epidemiology, Academy of Military Medical Sciences (Beijing, China).

### Sensitivity and Specificity when Testing Clinical Samples

A total of 99 samples including throat swab, sputum and fecal samples from 32 laboratory-confirmed H7N9 patients were tested in parallel with both real-time RT-PCR and our RDT immunoassay. The detection sensitivity of our immunoassay was 59.4% (19/32) for the 32 confirmed H7N9 patients. The highest detection rate, 61.5% (16/26), was achieved using sputum samples, while the detection sensitivity for nasopharyngeal swab and fecal samples was lower, at 35.7% (5/14) and 20.0% (2/10), respectively (Table 4). These were likely a result of the significantly higher virus titers found in sputum samples than nasopharyngeal swabs or fecal samples, as demonstrated by real-time RT-PCR. The average cycle threshold (Ct) values were 26.3±4.6 for sputum, 35.3±5.0 for nasopharyngeal swab and 35.5±5.3 for fecal samples (p<0.005) (Table 4).

**Table 4:** Rapid Diagnostic Test of clinical samples from influenza A(H7N9) positive patients.

Samples	No. of Patients	Ct of PCR	RDT detection	
			Positive No.	Positive rate (%)
Sputum	26	26.3±4.6	16	61.5
Throat swab	14	35.0±5.0	5	35.7
Feces	10	35.5±5.3	2	20.0

Our RDT assay achieved sensitivity of 33.3% (33/99) when testing 99 H7N9 positive clinical samples. The highest sensitivity was 84.6% (11/13) for samples positive at less than 26 Cts in real-time RT-PCR experiments, and declined with the increasing Cts required to detect HA by real-time RT-PCR. With samples positive at 27∼30 Cts, the RDT achieved sensitivity of 44.8% (13/29); with samples positive at 31∼35 Cts, the RDT achieved sensitivity of 24.5% (7/29); and with samples positive at >35 Cts, the RDT achieved lowest sensitivity of 7.1% (2/28) (Table 5). However, there was no false positive test result obtained when the throat swabs or sputum specimens of 180 patients without infection with H7N9 virus were tested (data not shown).

**Table 5:** Rapid Diagnostic Test and Real Time RT-PCR of clinical samples from influenza A(H7N9) positive patients.

Ct of Real-time RT-PCR	Samples tested (n)	RDT detection	
		Positive No.	Positive rate (%)
>35	28	2	7.1
31∼35	29	7	24.1
27∼30	29	13	44.8
≤26	13	11	84.6
Total	99	33	33.3

## Discussion

At present, the standard laboratory methods for testing for novel influenza A (H7N9) in China are virus isolation and real-time RT-PCR. However, these methods may take hours or days to produce results and require well trained personnel, sophisticated laboratory equipment and expensive reagents. The application of RDT in the wake of this outbreak would likely enhance disease surveillance and control.

In this study, we described the development and primary evaluation of influenza A H7-specific RDT for rapid detection of H7N9 virus infection. The RDT assay is based on the lateral flow platform using two specific H7-directed monoclonal antibodies for capture and detection of antigen. This immune assay is simple, easy to perform and can produce result in less than 15 min. Although the rapid immunoassay is less sensitive than real-time PCR, the RDT achieved an improved detection rate in samples with high levels of antigen.

Our study demonstrated that the H7-specific RDT could reach the LOD of 10^3^TCID_50_/ml or 30 Cts by real-time PCR. Among the 32 H7N9 patients confirmed to be H7N9 positive by RT-PCR, 59.4% (19/32) tested positive by our RDT. Furthermore, our RDT assay detected 33.3% (33/99) of H7N9 infections regardless the collection dates and clinical sample types. Within the low detection limit of about 30 Cts by real-time RT-PCR, our RDT assay achieved an overall sensitivity of 57.1% (24/42).

Baas et al has recently evaluated 6 commercially available RDTs by detecting nucleoprotein antigen of influenza viruses for their ability to detect H7N9 virus (A/Anhui/01/2013). They found that the LOD of five of the six RDTs for H7N9 virus ranged from 1x10^5^ to 1x10^5.5^ TCID_50_/mL or 22–24 Ct values, indicating less sensitive for H7N9 virus than for the seasonal or other influenza A(H7) viruses [16]. Taken Baas’s results and our study together, our H7 specific assay was more sensitive than the universal influenza A rapid assay for detection of H7N9 viruses.

Similar to the diagnosis of H5N1 infection, our study found that sputum samples may be more appropriate for diagnosing novel H7N9 virus infection and should be first tested if available [4]. This finding is consistent with the data suggesting that the current H7N9 epidemic often caused severe lower respiratory tract illness in humans, resulting in a relatively high concentration of virus in the sputum [1], [4]. Chen et al also reported that serial throat swab samples from one patient were consistently negative for H7N9 by real-time RT-PCR, but sputum samples were positive [4].

Our RDT did not give false positive results when recombinant antigens and inactive virus specimens of non-H7 subtypes of the influenza virus were probed, including H1, H3, H5, H9 subtypes. Furthermore, all throat swab and sputum clinical specimens from non-H7N9 patients also tested negative.

According to the best of our knowledge, this is the first rapid immunoassay that has been evaluated with H7N9 clinical samples for detection of HA antigen of H7 subtypes. This RDT can be used for rapid diagnosis and epidemiological study of H7N9 infection, which can be subsequently confirmed by virus culture and PCR.

Our study is limited by the small number of clinical samples from H7N9 patients because when we were developing the assay, less than 150 H7N9 patients have been confirmed H7N9 positive since the outbreak. Therefore, the detection sensitivity and specificity of this assay must be further evaluated when more H7N9 patient samples are available. In addition, our rapid assay can only detect HA antigen of H7 subtypes. For correct diagnosis and subtyping of H7N9 virus, neuraminidase (NA) detection should be integrated into the assay. The refinement and improvement of our RDT for detection of H7N9 virus are ongoing.

One interesting finding highlighted by this study is that the H7 directed monoclonal antibodies employed in this assay were very sensitive to and can specifically detect the H7N9 virus in clinical specimens. This observation raises the possibility of developing universal subtype specific assays using subtype specific antibodies. At present, 17 HA subtypes and 10 NA subtypes have been characterized, resulting in a total of 170 possible combinations of various HA and NA subtypes or recombinant influenza viruses [17]. If subtype specific assays could be developed and their usefulness could be approved, assays that are both sensitive and specific to possible new recombinant influenza viruses can be quickly developed in advance of their discovery. Such assays would provide important tools for prevention and control of new influenza epidemics.
